# Formation-Constrained Cooperative Localization for UAV Swarms in GNSS-Denied Environments

**DOI:** 10.3390/s26061984

**Published:** 2026-03-22

**Authors:** Qin Li, Peng Wang, Xiaochun Li, Jieyong Zhang, Ying Luo, Wangsheng Yu, Haiyan Cheng

**Affiliations:** College of Information and Navigation, Air Force Engineering University, Xi’an 710077, China; blueking1985@hotmail.com (P.W.); chunwind@sohu.com (X.L.); dumu3110728@126.com (J.Z.); luoying2002521@163.com (Y.L.); xing_fu_yu@sina.com (W.Y.); chenghaiyan78@126.com (H.C.)

**Keywords:** cooperative localization, formation constraints, UAV swarm, GNSS-denied environments, backbone-listener scheme

## Abstract

Cooperative localization is critical for UAV swarm operations in GNSS-denied environments. The backbone-listener scheme, using a small subset of agents as active backbone nodes and others as passive listeners, offers notable advantages in reducing communication overhead and enhancing swarm scalability. Building on this scheme, we propose a formation-constrained cooperative localization method to improve accuracy by integrating known formation geometry into the localization process. First, backbone node selection uses a formation-constrained greedy node activation (GNA) strategy with weighted distance fusion, combining measured and ideal formation distances to enable near-optimal selection aligned with formation structure. Second, listener node localization incorporates formation constraints into Chan’s algorithm, paired with angle-of-arrival (AOA) refinement, to ensure estimated positions match expected inter-agent distances. Third, global optimization uses a gradient descent-based refinement to enforce formation constraints across all agent positions. Our theoretical derivations and simulations are limited to the two-dimensional (2D) case. Simulation results validate the proposed method’s improved success rate, reliability, and stability. Its effectiveness is demonstrated across various formation types, with robust adaptability to asymmetric geometries shown to be a valuable feature for practical deployment.

## 1. Introduction

With the continuous development of swarm control technologies and the advancement of fields such as low-altitude economy and intelligent infrastructure monitoring, multi-UAV swarms have become a key platform for transforming aerial operations [1,2]. Their application value is increasingly prominent in scenarios such as full-domain reconnaissance, disaster rescue, and collaborative power grid inspection [3]. Accurate positioning and navigation capabilities are the key prerequisite for efficient collaborative operations of UAV swarms [4]. Currently, mainstream solutions mainly rely on the Global Navigation Satellite System (GNSS) to obtain position information. However, in complex environments like urban canyons, mountain gullies, and electromagnetically jammed battlefields, GNSS signals tend to lose lock. This is caused by blockage, multipath reflection, or intentional jamming, thus leading to problems such as swarm positioning drift, interrupted collaborative tasks, and even UAV collisions [5,6]. Cooperative localization has emerged as a promising solution for GNSS-denied scenarios [7]. Ultra-Wideband (UWB) technology offers high-precision ranging capability (centimeter-level positioning accuracy) due to its ultra-wide operating bandwidth. It also has strong resistance to multipath interference and electromagnetic interference, as well as excellent adaptability to non-line-of-sight (NLOS) environments. Thus, it has become the preferred technology for UAV swarm positioning in GNSS-denied environments [8,9,10,11].

Cooperative localization for multi-UAV systems has received significant attention. Existing localization algorithms can be classified into three categories: anchor node-based, multidimensional scaling (MDS)-based, and distributed cooperative methods.

Anchor node-based positioning algorithms achieve target position estimation by deploying fixed, known-position anchor nodes to establish a local positioning network. Their core mechanism relies on obtaining signal parameters between unknown nodes (target) and anchor nodes through radio frequency (RF) communications—including Time of Arrival (TOA), Time Difference of Arrival (TDOA), and Angle of Arrival (AOA)—to estimate positions via triangulation or hyperbolic methods. This type of algorithm has several typical variants for different application scenarios: TOA/TDOA/AOA localization algorithms use a single signal metric, with simple principles but poor resistance to multipath noise interference, thus requiring high-density anchor nodes to guarantee accuracy [4,12,13,14,15]. The Chan algorithm improves TDOA localization accuracy via two-step least squares estimation, with high computational efficiency [16]. Hybrid TDOA/AOA localization algorithms combine time difference and angle information to reduce the localization ambiguity of single-metric algorithms, thus improving stability under non-line-of-sight (NLOS) conditions [17]. A common limitation of such algorithms is the high cost of anchor node deployment. Moreover, fixed anchor nodes cannot dynamically follow mobile multi-agents, making them difficult to adapt to dynamically changing network topologies and limiting their applicability in scenarios such as UAV swarms.

To reduce dependence on anchor nodes, MDS-based positioning algorithms are developed. They do not require fixed anchor nodes, construct a similarity matrix based on inter-node relative measurements (e.g., distance or angle), and recover relative positions via MDS, serving as a core solution for infrastructure-free localization. Classical MDS (C-MDS) constitutes the foundation of such algorithms and achieves position reconstruction with high accuracy via distance measurements in fully connected networks [18]. Nyström approximation-based MDS reduces complexity via node sampling, balancing accuracy and efficiency [19]. Hierarchical MDS employs network clustering to enable distributed computation, thereby reducing centralized bottlenecks [20]. Distributed noise-robust MDS adds noise suppression to reduce error propagation [21]. Three-dimensional distributed MDS extends to 3D space for UAV networks [22]. Complex MDS optimizes nonlinear model solution efficiency for wide-band and mobile scenarios [23]. Continuous optimization has addressed issues of complexity, robustness, and dimension adaptation, making it the preferred solution for infrastructure-free scenarios.

While MDS-based algorithms are the preferred choice for infrastructure-free localization via continuous optimization, their applicability in large-scale UAV swarms is limited by the inherent need for full-mesh communication. This causes excessive overhead, conflicting with the real-time requirements of swarm cooperative localization and spurring the development of distributed cooperative localization algorithms.

Distributed cooperative localization algorithms are specifically designed for distributed multi-agent networks, achieving global relative positioning through local inter-node communication and cooperation without a centralized processing unit [24]. Guo et al. proposed a UWB-IMU fusion method for multi-UAV positioning, reducing anchor node dependence [25]. Lv et al. proposed a UWB-based distributed Kalman filtering method for cooperative localization, enabling high-precision positioning in partially GNSS-denied environments [26]. Additionally, the backbone-listener localization algorithm proposed in recent studies selects backbone nodes via the GNA strategy, enables passive localization of listener nodes, and optimizes accuracy through back calibration. This distributed architecture achieves decimeter-level accuracy and supports large-scale network expansion [27].

Distributed cooperative localization algorithms have addressed the limitations of anchor node-based and MDS-based methods, with promising performance in UAV swarms. However, for complex swarm operations, conventional methods fail to sufficiently exploit formation geometry information as complementary prior knowledge, limiting accuracy and robustness in dynamic conditions. Notably, in practical UAV swarm operations, the formation geometry, as a basic constraint for mission execution, contains valuable spatial correlation information that has not been sufficiently integrated into localization optimization, while extensive literature has focused on formation control [28,29], few existing studies utilize relevant formation information in localization. Specifically, Schindler et al. developed a relatively infrastructure-free localization algorithm for swarm formations [30], yet their work lacks the explicit incorporation of formation constraints as a priori knowledge. This reflects a broader research gap wherein formation control and localization have traditionally been investigated as isolated research domains, with insufficient attention paid to the cross-integration of formation-derived information into localization processes.

This gap motivates the need for a systematic approach that explicitly incorporates formation constraints as a priori knowledge in the localization optimization process. This work aims to add to existing research by directly integrating a priori formation geometric constraints into cooperative localization optimization, thereby seeking to develop a unified optimization framework that may jointly improve localization accuracy and robustness. In practical UAV swarm operations, maintaining predetermined formation patterns (e.g., linear, circular, or grid formation) is a basic requirement for mission execution; when appropriately used as an a priori constraint, such formation-related geometric information may provide supplementary spatial correlation indicators to help reduce localization ambiguity. The primary contributions of this work are outlined as follows:

(1) A formation-constrained greedy node activation (GNA) strategy is proposed, which integrates formation geometry information through weighted distance fusion to improve backbone node selection and may enable near-optimal selection that aligns with the formation structure.

(2) A formation-constrained listener localization method is proposed, which integrates formation constraints into Chan’s algorithm with angle-of-arrival (AOA) refinement, helping ensure estimated positions conform to expected inter-agent distances.

(3) A global formation constraint optimization stage is proposed, which uses gradient descent-based refinement to strengthen formation constraints across all agent positions after initial localization.

The remainder of this paper is organized as follows: Section 2 formulates the problem. Section 3 presents the cooperative localization algorithm with formation constraints. Section 4 analyzes theoretical aspects of the proposed method. Section 5 presents numerical experiments. Section 6 concludes the paper.

## 2. Problem Formulation

This section introduces the network setting with a backbone-listener scheme, explains the measurement model, and formulates the cooperative localization problem with formation constraints. Table 1 summarizes the mathematical notation used throughout this section and the remainder of the paper.

### 2.1. Network Setting

We consider a two-dimensional (The present analysis is confined to the planar case for clarity. Extension to 3D requires augmenting the state and AOA models; the main algorithmic structure remains applicable, with limitations discussed in the conclusion) network that consists of *N* agents, whose set is denoted as N={1,2,…,N}. For agent i∈N, the global position is denoted as $p_{i}={\left[x_{i},y_{i}\right]}^{T}\in R^{2}$, and the set of its neighbor agents is denoted as $N_{i}\subseteq N$, with $|N_{i}|=N_{i}$ elements. In the local coordinate system of agent *i*, the relative position of any other agent $j\in N_{i}$ is $p_{i\leftarrow j}={\left[x_{j}-x_{i},\;y_{j}-y_{i}\right]}^{T}$. The relative position parameter of the formation around agent *i* is then denoted as $P_{i\leftarrow N_{i}}={\left[\ldots ,p_{i\leftarrow j},\ldots \right]}_{j\in N_{i}}$.

Additionally, the global orientation parameter vector is denoted as $\alpha ={\left[\alpha_{1},\alpha_{2},\ldots ,\alpha_{N}\right]}^{T}$, where $\alpha_{i}$ is the orientation of agent *i*. The global position parameter vector is defined as $p={\left[p_{1}^{T},p_{2}^{T},\ldots ,p_{N}^{T}\right]}^{T}$, and the estimations of α and p are denoted as $\hat{\alpha }$ and $\hat{p}$, respectively.

**Figure 1:**
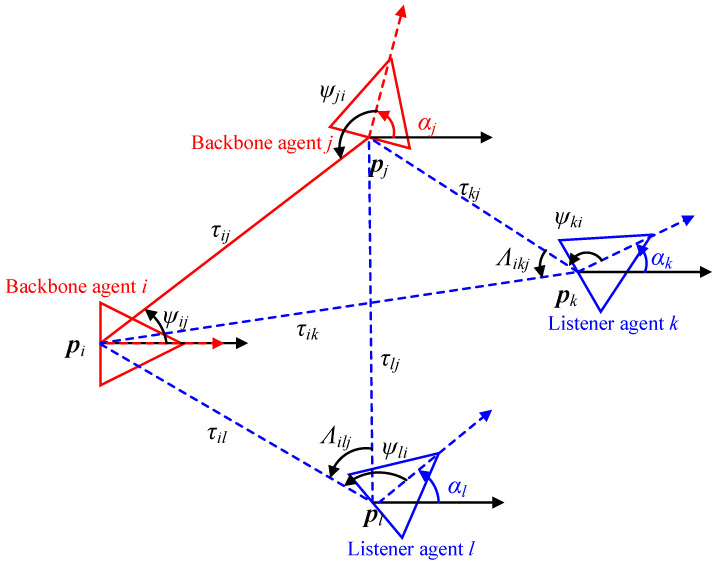
Illustration of the backbone-listener scheme, where two red backbone agents *i* and *j* transmit signals and two blue listener agents *k* and *l* only receive signals.

The network adopts a backbone-listener scheme [27]. Backbone nodes actively transmit and receive wireless signals to establish mutual constraints; listener nodes passively receive signals for localization, avoiding redundant communication. The set of $N_{b}$ backbone agents is denoted as $N_{b}$, with $|N_{b}|=N_{b}$, and the set of $N_{L}$ listener agents as $N_{L}$, with $|N_{L}|=N_{L}=N-N_{b}$. The scheme is illustrated in Figure 1. Roles may be fixed or switched; backbone selection can be preset or determined by a node activation strategy (see Section 3.1).

### 2.2. Measurement Model

Agent $j\in N_{i}$ transmits a wideband signal to agent *i*. Agent *i* receives the signal and measures the Time of Arrival (TOA) and Angle of Arrival (AOA).

**TOA Measurement:** The measured distance between agent *i* and agent *j* is

(1) $${\tilde{d}}_{ij}=d_{ij}+n_{\tau ,ij}\cdot c,$$

where *c* is the speed of signal, and $n_{\tau ,ij}∼N\left(0,\sigma_{\tau }^{2}\right)$ is the time measurement noise. The TOA measurement is

(2) $$\tau_{ij}=\frac{{\tilde{d}}_{ij}}{c}=\frac{d_{ij}}{c}+n_{\tau ,ij}.$$

For each time slot, the distance between agent *i* and agent *j* is:

(3) $$d_{ij}=∥p_{i}-p_{j}∥=\sqrt{{\left(x_{i}-x_{j}\right)}^{2}+{\left(y_{i}-y_{j}\right)}^{2}}.$$

The unknown parameter ***θ*** in the localization problem is   

(4) $$\theta ={\left[\theta_{1}^{T},\theta_{2}^{T},\ldots ,\theta_{N}^{T}\right]}^{T},$$

where $\theta_{i}={\left[p_{i}^{T},\alpha_{i}\right]}^{T}$, i∈N.

**AOA Measurement:** Note that the orientation α and the AOA measurement ψ are different parameters, as shown in Figure 1. The AOA measurement model differs for backbone and listener agents due to their different roles in the network.

For backbone agents, both sides of AOA measurements are available; thus, the relationship between orientation and AOA is given by

(5) $$\alpha_{i}+\psi_{ij}\equiv \alpha_{j}+\psi_{ji}+\pi \;\left(\mathrm{mod}\;2\pi \right),\;i,j\in N_{b}$$

where $\psi_{ij}$ is the AOA measurement at agent *i* from agent *j*, and $\alpha_{i}$ is the orientation of agent *i*.

For listener agents, only one direction of AOA is available. As illustrated in Figure 1, when listener agent $k\in N_{L}$ receives a signal from backbone agent $i\in N_{b}$, the AOA measurement $\psi_{ki}$ is available. However, the measurement $\psi_{ik}$ is missing. Therefore, the estimation of the orientation and position for listener agents is obtained based on the listener’s position estimation ${\hat{p}}_{k}$, which is obtained by

(6) $$\alpha_{k}+\psi_{ki}=\arctan\left(\frac{y_{i}-{\hat{y}}_{k}}{x_{i}-{\hat{x}}_{k}}\right),$$

where ${\hat{p}}_{k}={\left[{\hat{x}}_{k},{\hat{y}}_{k}\right]}^{T}$ is the estimated position of listener agent *k*.

Then, the cooperative localization problem is formulated as

(7) $$\min_{\theta }J\left(\theta \right)=\sum_{i=1}^{N}\sum_{j\in N_{i}}\left[{\left(\frac{{\hat{d}}_{ij}}{c}-\tau_{ij}\right)}^{2}+{\left({\hat{\psi }}_{ij}-\psi_{ij}\right)}^{2}\right],$$

where ${\hat{d}}_{ij}$ and ${\hat{\psi }}_{ij}$ are the predicted range and bearing between agents *i* and *j*.

## 3. Cooperative Localization with Formation Constraints

This section presents the cooperative localization algorithm with formation constraints based on the backbone-listener scheme. We first introduce the formation-constrained greedy node activation strategy for activating backbone nodes. Subsequently, we perform localization of backbone and listener nodes based on the backbone-listener scheme, with a detailed description of the formation-constrained listener localization method.

### 3.1. Formation-Constrained Greedy Node Activation (GNA)

The selection of backbone agents can be formulated as an optimization problem that aims to minimize the relative localization error of the network. Directly minimizing this error is a non-convex problem, and convex-relaxation-based approximations tend to be computationally intensive for practical swarm systems. Reference [27] adopts a suboptimal yet computationally efficient GNA strategy, which follows two principles: (1) the localization performance degrades when listener agents lie outside the convex hull formed by the transmitting agents and (2) the direction of the received signal affects the major axis of the “information ellipse,”; thus, backbone agents should be placed as dispersively as possible around the listener agents to maximize information gain.

The standard GNA relies only on noisy measured distances, which may not reflect the actual formation structure. To address this limitation, this work proposes a formation-constrained GNA strategy that integrates formation geometry information to select more suitable backbone nodes. To achieve this integration, this work introduces a weighted distance fusion approach that fuses measured distances with ideal inter-agent distances. The core insight is that the measured distance $d_{ij}$ (from noisy measurements) may be unreliable, while the ideal inter-agent distance $d_{ij}^{f}$ (derived from prior formation knowledge) reflects the intrinsic geometric potential. Thus, this work fuses these two distance estimates through a weighted fusion equation,

(8) $$d_{ij}^{\mathrm{fused}}=\left(1-\gamma \left(\lambda \right)\right)\cdot d_{ij}+\gamma \left(\lambda \right)\cdot d_{ij}^{f},$$

where $d_{ij}$ is the measured distance between agents *i* and *j*, and $d_{ij}^{f}$ is the expected formation distance. The adaptive weight γ(λ)∈[0,1] is defined as

(9) $$\gamma \left(\lambda \right)=\frac{\lambda }{\lambda +\lambda_{0}},$$

where $\lambda_{0}>0$ is a threshold parameter that determines when formation information becomes dominant. When λ is small (weak formation constraints), γ(λ)≈0 and the algorithm behaves like standard GNA. When λ is large (strong formation constraints), γ(λ)≈1 and formation geometry plays a more significant role. The fused distance $d_{ij}^{\mathrm{fused}}$ provides a more reliable estimate that utilizes both measurement data and formation prior knowledge. When measurement noise causes $d_{ij}$ to deviate significantly, the ideal distance $d_{ij}^{f}$ pulls it back to the true geometric level, preventing the GNA algorithm from being misled by noisy measurements.

The improved potential field function is then computed based on the fused distance

(10) $$f_{d}\left(d_{ij},d_{ij}^{f},\lambda \right)=\frac{1}{\beta_{d}d_{ij}^{\mathrm{fused}}},$$

where $\beta_{d}>0$ is a scaling parameter.

The formation-constrained GNA algorithm is shown in Algorithm 1.
**Algorithm 1** Formation-constrained Greedy Node Activation1:**Input:** $N_{b}$, $p_{i}$ (i∈N), λ, $\lambda_{0}$, $D_{f}$2:**Output: **$N_{b}$3:Compute $\gamma =\lambda /(\lambda +\lambda_{0})$ and $D_{\mathrm{real}}$ from $p_{i}$4:Initialize $N_{b}^{\mathrm{best}}=\emptyset$, $P_{\min}=\infty$5:**for** each starting agent i∈N **do**6:   Compute fused distances from *i*: $d_{ik}^{\mathrm{fused}}=\left(1-\gamma \right)\cdot {\left[D_{\mathrm{real}}\right]}_{ik}+\gamma \cdot {\left[D_{f}\right]}_{ik}$, ∀k∈N7:   Find $j=\arg\min_{k\in N}f_{d}\left(d_{ik}^{\mathrm{fused}}\right)$; compute $d_{jk}^{\mathrm{fused}}$ from *j* and find $k_{1}=\arg\min_{k\in N}f_{d}\left(d_{jk}^{\mathrm{fused}}\right)$8:   Initialize $N_{b}=\left\{k_{1}\right\}$, n=29:   **while** $n\leq N_{b}$ **do**10:     Find $k_{n}=\arg\min_{k\in N\setminus N_{b}}\sum_{j\in N_{b}}f_{d}\left(d_{kj}^{\mathrm{fused}}\right)$ and add $k_{n}$ to $N_{b}$11:     Set n=n+112:   **end while**13:   Compute $P=\sum_{a=1}^{N_{b}}\sum_{b=a+1}^{N_{b}}f_{d}\left(d_{ab}^{\mathrm{fused}}\right)$14:   **if** $P<P_{\min}$ **then**15:     Update $P_{\min}\leftarrow P$, $N_{b}^{\mathrm{best}}\leftarrow N_{b}$16:   **end if**17:**end for**18:Return $N_{b}=N_{b}^{\mathrm{best}}$

**Remark** **1.** *Formation constraints and GNA are complementary: the combination yields more consistent measurement configurations that match the expected formation geometry; additional constraints from known inter-agent distances help resolve localization ambiguities, especially under sparse or noisy measurements.*

### 3.2. Backbone Relative Localization

The backbone agents are localized relative to each other using the classical multidimensional scaling (C-MDS) method. The position parameter of backbone agents is denoted as $P_{N_{b}}\in R^{2\times N_{b}}$.

For a selected agent $i\in N_{b}$, the coordinate system is established by finding the agent *j* closest to it and setting the direction from *i* to *j* as the *x*-axis. According to (5), this implies

(11) $$\alpha_{ij}=-\psi_{ij},\;\alpha_{ji}=\pi -\psi_{ji}.$$

With backbone agents in signal-transmitting mode, the TOA and AOA measurements are collected as

(12) $$\begin{array}{cc} \tau_{i\leftarrow N_{b}} & ={\left[\ldots ,\tau_{ij},\ldots \right]}^{T}, \end{array}$$

(13) $$\begin{array}{cc} \Psi_{i\leftarrow N_{b}} & ={\left[\ldots ,\psi_{ij},\ldots \right]}^{T},\;j\in N_{b}. \end{array}$$

The distance matrix is constructed as $D_{i\leftarrow N_{b}}=\mathrm{diag}\left\{c\tau_{i\leftarrow N_{b}}\right\}$. The angle between links from $j_{1}$ and $j_{2}$ towards *i* is

(14) $$A_{j_{1}j_{2}}^{i}=\psi_{ij_{1}}-\psi_{ij_{2}},\;j_{1},j_{2}\in N_{b}.$$

The angle-based correlation matrix $A_{i}\in R^{N_{b}\times N_{b}}$ is constructed as

(15) $$A_{i}=\left[\begin{array}{cccc} 1 & \cos A_{j_{1}j_{2}}^{i} & \ldots & \cos A_{j_{1}N_{b}}^{i}\\ \cos A_{j_{2}j_{1}}^{i} & 1 & \ldots & \cos A_{j_{2}N_{b}}^{i}\\ \vdots & \vdots & \ddots & \vdots \\ \cos A_{N_{b}j_{1}}^{i} & \cos A_{N_{b}j_{2}}^{i} & \ldots & 1 \end{array}\right].$$

Performing singular value decomposition (SVD) of $A_{i}$ yields $A_{i}=U_{i}\Sigma_{i}U_{i}^{T}$, where $U_{i}$ contains the left singular vectors and $\Sigma_{i}$ is a diagonal matrix of singular values. The relative positions of other agents with respect to agent *i* are estimated as

(16) $${\hat{P}}_{i\leftarrow N_{b}}=\left[\left(U_{i}\sqrt{\Sigma_{i}}\right)D_{1:2,1:N_{b}-1}^{T}\right],$$

where $D_{1:2,1:N_{b}-1}$ denotes the first two rows and first $N_{b}-1$ columns of the distance matrix $D_{i\leftarrow N_{b}}$.

The algorithm above is derived under the assumption that there is no noise in the measurement; however, it can still perform estimation in the presence of noise.

### 3.3. Listener Relative Localization with Formation Constraint

Under the backbone-listener scheme (Section 2.1), listener agents rely on backbone nodes for passive localization. As noted in Section 2.2, $\psi_{ik}$ is unavailable for listeners; hence, it cannot be estimated from (5). The listener relative localization algorithm therefore proceeds as follows. First, Chan’s algorithm [16] obtains an initial position estimate based on TOA measurements. Then, the localization result is refined by incorporating AOA information and formation constraints derived from the initial solution. The detailed procedure is described below.

#### 3.3.1. Listener Relative Localization

Let ${\bar{N}}_{k}\subseteq N_{b}$ denote the set of backbone neighbors of listener agent *k*, with ${\bar{N}}_{k}=\left|{\bar{N}}_{k}\right|$. The initial position $p_{k}$ is estimated as ${\hat{p}}_{k}^{\left(0\right)}$ using the Chan algorithm [16]. For listener agent *k* with ${\bar{N}}_{k}$ backbone neighbors, Chan’s algorithm constructs a linear system from TOA measurements. The measurement matrix $G^{\left(0\right)}$ and measurement vector $h^{\left(0\right)}$ are derived from the distance measurements between listener *k* and its backbone neighbors. The initial position estimate is obtained by solving

(17) $${\hat{p}}_{k}^{\left(0\right)}=\arg\min_{p_{k}}{\left(h^{\left(0\right)}-G^{\left(0\right)}p_{k}\right)}^{T}{\left(\Psi^{\left(0\right)}\right)}^{-1}\left(h^{\left(0\right)}-G^{\left(0\right)}p_{k}\right),$$

where $\Psi^{\left(0\right)}$ is the weighted covariance matrix. Solving (17) yields an initial estimated position ${\hat{p}}_{k}^{\left(0\right)}$, which is used to calculate the initial orientation ${\hat{\alpha }}_{k}^{\left(0\right)}$ through the geometric relationship in (6). The weighted covariance matrix is computed as

(18) $$\Psi^{\left(0\right)}=c^{2}D_{k\leftarrow {\bar{N}}_{k}}QD_{k\leftarrow {\bar{N}}_{k}}^{T},$$

where $D_{k}=D_{k\leftarrow {\bar{N}}_{k}}$ is a diagonal distance matrix with elements ${\left[D_{k}\right]}_{ii}={\tilde{d}}_{ik_{i}}$ (the measured distance from listener *k* to backbone neighbor $k_{i}$), and $Q=\mathrm{diag}\{{\left[\sigma_{\tau ,k_{2}}^{2},\ldots ,\sigma_{\tau ,k_{{\bar{N}}_{k}}}^{2}\right]}^{T}\}$ is the noise covariance matrix with $\sigma_{\tau ,k_{j}}^{2}$ being the variance of the TOA measurement noise.

Based on the initial estimation ${\hat{p}}_{k}^{\left(0\right)}$ and estimated orientation ${\hat{\alpha }}_{k}^{\left(0\right)}$, AOA information is used to refine the position estimation. According to (6), by linearizing the geometric relationships and applying appropriate approximations, the augmented measurement model becomes

(19) $$G_{k}^{*}=\left[\begin{array}{c} G^{\left(0\right)}\\ {}[-\sin\left(\psi_{k,1}\right),\cos\left(\psi_{k,1}\right),0]\\ \vdots \\ {}[-\sin\left(\psi_{k,{\bar{N}}_{k}}\right),\cos\left(\psi_{k,{\bar{N}}_{k}}\right),0] \end{array}\right],\;h_{k}^{*}=\left[\begin{array}{c} h^{\left(0\right)}\\ 0_{{\bar{N}}_{k}} \end{array}\right],$$

where $G_{k}^{*}\in R^{(2{\bar{N}}_{k}-1)\times 2}$ is the augmented measurement matrix combining TOA and AOA measurements, $h_{k}^{*}$ is the augmented measurement vector, ${\bar{N}}_{k}$ is the number of backbone neighbors of listener agent *k*, and $\psi_{k,j}$ denotes the AOA measurement from listener *k* to backbone agent *j*. The covariance matrix $\Psi^{*}$ is updated as

(20) $$\Psi^{*}=\mathrm{diag}\left\{{\left[\Psi^{\left(0\right)},\sigma_{\psi ,k_{1}}^{2},\ldots ,\sigma_{\psi ,k_{{\bar{N}}_{k}}}^{2}\right]}^{T}\right\},$$

where $\sigma_{\psi ,k_{j}}^{2}$ is the variance of the AOA measurement noise from listener *k* to backbone neighbor *j* (indexed by $j\in \{1,\ldots ,{\bar{N}}_{k}\}$). The refined position estimate is then obtained by

(21) $${\hat{p}}_{k}=\arg\min_{p_{k}}{\left(h_{k}^{*}-G_{k}^{*}p_{k}\right)}^{T}{\left(\Psi^{*}\right)}^{-1}\left(h_{k}^{*}-G_{k}^{*}p_{k}\right).$$

The orientation estimation can be updated using ${\hat{p}}_{k}$ through the geometric relationship in (6).

#### 3.3.2. Formation-Constrained Listener Localization

When formation constraints are enabled, the constraint terms are incorporated into the optimization. For listener agent *k*, the formation-constrained refinement extends the augmented system in (19) as:

(22) $$\left[\begin{array}{c} G_{k}^{*}\\ \lambda_{1}C_{f} \end{array}\right]p_{k}=\left[\begin{array}{c} h_{k}^{*}\\ \lambda_{1}d_{f} \end{array}\right],$$

where $C_{f}$ is the linearized formation constraint matrix for listener agent *k*, $d_{f}$ is the corresponding constraint vector containing expected formation distances, and $\lambda_{1}>0$ is a constraint weight parameter for listener localization. The formation-constrained position estimate is then obtained by solving:

(23) $$\begin{array}{cc} {\hat{p}}_{k}^{\mathrm{fc}} & =\arg\min_{p_{k}}\left[{\left(h_{k}^{*}-G_{k}^{*}p_{k}\right)}^{T}{\left(\Psi^{*}\right)}^{-1}\right.\\ \; & \;\left.\times \left(h_{k}^{*}-G_{k}^{*}p_{k}\right)+\lambda_{1}J_{f}\left(p_{k}\right)\right], \end{array}$$

where $J_{f}\left(p_{k}\right)$ is the formation constraint term for agent *k*.

The formation structure is represented by a distance matrix $D_{f}\in R^{N\times N}$, where ${\left[D_{f}\right]}_{ij}=d_{ij}^{f}$ denotes the expected distance between agent *i* and agent *j* in the formation. The formation constraint loss is unified as:

(24) $$J_{f}\left(p\right)=\sum_{i=1}^{N}\sum_{j\in N_{i},j\neq i}\frac{1}{2}{\left(∥p_{i}-p_{j}∥-d_{ij}^{f}\right)}^{2},$$

where $d_{ij}^{f}>0$ if agents *i* and *j* have a formation relationship, and $d_{ij}^{f}=0$ otherwise.

### 3.4. Back Calibration

After listener localization (Section 3.3), backbone positions are refined using measurements from listener agents (Section 2.1), improving overall accuracy [27].

For a listener agent *k*, let ${\hat{p}}_{{\bar{N}}_{k}}$ denote the estimated positions of its backbone agent neighbors. Let $\Psi_{k}$ and $\Psi_{{\bar{N}}_{k}}$ be the covariance matrices for agent *k* and its backbone neighbors, respectively.

Then, a linear back calibration step is written as

(25) $${\tilde{p}}_{{\bar{N}}_{k}}=Y_{p}{\hat{p}}_{{\bar{N}}_{k}}+Y_{z}m_{z,k},$$

where $m_{z,k}$ stacks the range and AOA measurements between listener *k* and its backbone neighbors as defined in Section 2.2 (with entries ${\tilde{d}}_{ki}$, $\psi_{ki}$, etc.), and $Y_{p}$ and $Y_{z}$ are parameter matrices with appropriate dimensions to be solved.

A first-order approximation of the measurement model in Section 2.2 around the current estimates yields an equivalent linear model

(26) $$m_{z,k}=H_{z,k}p_{{\bar{N}}_{k}}+n_{z,k},$$

where $H_{z,k}=\frac{\partial m_{z,k}}{\partial p_{{\bar{N}}_{k}}}$ is the Jacobian matrix, and $n_{z,k}∼N\left(0,\Psi_{R_{k}}\right)$. Here, $\Psi_{R_{k}}$ accounts for both measurement noise and the uncertainty of the listener estimate via error propagation

(27) $$\Psi_{R_{k}}=\Psi_{\mathrm{meas},k}+H_{k}\Psi_{k}H_{k}^{T},$$

where $\Psi_{\mathrm{meas},k}$ is the measurement noise covariance of $m_{z,k}$, and $H_{k}=\frac{\partial m_{z,k}}{\partial \theta_{k}}$ is the Jacobian with respect to the listener state $\theta_{k}$ (Section 2.2).

The goal of back calibration is to minimize the mean squared error (MSE) of backbone agents. The optimization problem can be expressed as

(28) $$\min_{Y_{p},Y_{z}}E\left\{∥Y_{p}{\hat{p}}_{{\bar{N}}_{k}}+Y_{z}m_{z,k}-p_{{\bar{N}}_{k}}{∥}^{2}\right\}.$$

Solving (28) by taking derivatives with respect to $Y_{p}$ and $Y_{z}$ and setting them to zero, we obtain the closed-form solution

(29) $$\begin{array}{cc} Y_{z} & =\Psi_{{\bar{N}}_{k}}H_{z,k}^{T}{\left(H_{z,k}\Psi_{{\bar{N}}_{k}}H_{z,k}^{T}+\Psi_{R_{k}}\right)}^{-1}, \end{array}$$

(30) $$\begin{array}{cc} Y_{p} & =I_{2|{\bar{N}}_{k}|}-Y_{z}H_{z,k}, \end{array}$$

Substituting $Y_{p}$ and $Y_{z}$ back into (25) yields the calibrated coordinates of the backbone agents.

Next, we update the orientation estimation of backbone agent $i\in {\bar{N}}_{k}$. For such *i*, let ${\bar{N}}_{i}=N_{i}\cap N_{b}$ denote the set of its backbone neighbors. Using the calibrated positions ${\tilde{p}}_{{\bar{N}}_{k}}$ and the geometric relationship in (5), the preliminary orientation estimation is

(31) $${\hat{\alpha }}_{i|j}=\arctan\left({\tilde{y}}_{j}-{\tilde{y}}_{i},\;{\tilde{x}}_{j}-{\tilde{x}}_{i}\right)-\psi_{ij},\;j\in {\bar{N}}_{i},$$

where $\left({\tilde{x}}_{i},{\tilde{y}}_{i}\right)$ and $\left({\tilde{x}}_{j},{\tilde{y}}_{j}\right)$ are the corresponding elements in ${\tilde{p}}_{{\bar{N}}_{k}}$. Combined with the geometric relationship (5), a set of equations can be obtained

(32) $$\left\{\begin{array}{c} \alpha_{i}-{\hat{\alpha }}_{i|j}=0\\ \alpha_{i}+\psi_{ij}=\alpha_{j}+\psi_{ji}+\pi \;\left(\mathrm{mod}\;2\pi \right) \end{array}\right.\;i\in {\bar{N}}_{k},j\in {\bar{N}}_{i}.$$

Solving (32) by the least squares method, we obtain the calibrated orientation ${\tilde{\alpha }}_{i}$ of each backbone agent.

### 3.5. Formation Constraint Optimization

Finally, a global formation constraint optimization is performed to ensure consistency with the known formation geometry.

The formation constraint optimization problem is

(33) $$\min_{\hat{p}}\;J_{f}\left(\hat{p}\right)=\sum_{i=1}^{N}\sum_{j\in N_{i},j\neq i}\frac{1}{2}{\left(∥{\hat{p}}_{i}-{\hat{p}}_{j}∥-d_{ij}^{f}\right)}^{2},$$

subject to maintaining reasonable agreement with the measurement-based estimates.

The optimization uses gradient descent with the following update rule.

**Gradient Computation:** For distance-based formation constraints, the gradient with respect to agent *i*’s position is:

(34) $$\frac{\partial J_{f}}{\partial {\hat{p}}_{i}}=\sum_{j\in N_{i},j\neq i}\left(∥{\hat{p}}_{i}-{\hat{p}}_{j}∥-d_{ij}^{f}\right)\cdot \frac{{\hat{p}}_{i}-{\hat{p}}_{j}}{∥{\hat{p}}_{i}-{\hat{p}}_{j}∥},$$

provided $∥{\hat{p}}_{i}-{\hat{p}}_{j}∥>\epsilon$ for a small threshold ϵ>0.

**Update Rule:**

(35) $${\hat{p}}_{i}^{\left(k+1\right)}={\hat{p}}_{i}^{\left(k\right)}-\alpha \lambda_{2}\frac{\partial J_{f}}{\partial {\hat{p}}_{i}}{|}_{{\hat{p}}^{\left(k\right)}},$$

where α>0 is a learning rate and $\lambda_{2}$ is a formation constraint weight for post-processing optimization.

**Convergence Criterion:** The algorithm terminates when the change between two consecutive iterations becomes sufficiently small, i.e.,

(36) $$∥{\hat{p}}^{\left(k+1\right)}-{\hat{p}}^{\left(k\right)}{∥}_{F}<\epsilon_{\mathrm{tol}},$$

where $\epsilon_{\mathrm{tol}}>0$ is a prescribed tolerance. A maximum iteration limit $K_{\max}$ is also enforced to avoid excessive computation.

The overall derivation of the formation-constrained cooperative localization algorithm with optimization is given in Algorithm 2.
**Algorithm 2** Formation-Constrained Cooperative Localization Algorithm1:**Input:** All agents N; TOA measurements $\tau_{ij}$; AOA measurements $\psi_{ij}$; formation matrix $D_{f}$; constraint weights $\lambda_{1}$, $\lambda_{2}$2:**Output:** Estimated positions ${\hat{p}}_{i}$ for all agents i∈N3:**GNA:** Select $N_{b}$ backbone agents by minimizing potential field (see Algorithm 1)4:**Backbone Localization:** Given backbone set $N_{b}$ and distance matrix D, use MDS to localize backbone agents relative to each other and establish reference frame5:**for** each listener agent $k\in N_{L}$ **do**6:   **Step 1—Chan Algorithm:** Solve (17) using TOA measurements to obtain initial position estimate ${\hat{p}}_{k}^{\left(0\right)}$ and orientation ${\hat{\alpha }}_{k}^{\left(0\right)}$7:   **Step 2—AOA Refinement:** Construct augmented system $G_{k}^{*}$ and $h_{k}^{*}$ as in (19), update covariance $\Psi^{*}$ as in (20), and solve (21) to obtain refined position ${\hat{p}}_{k}$8:   **if** formation constraints enabled **then**9:     **Formation-Constrained Refinement:** Extend the augmented system as in (22) and solve (23) to obtain formation-constrained position estimate ${\hat{p}}_{k}^{\mathrm{fc}}$10:   **end if**11:**end for**12:**Back Calibration:** For each listener *k*, form $m_{z,k}$ and Jacobians $H_{z,k}$ and $H_{k}$, compute $\Psi_{R_{k}}$, then update ${\tilde{p}}_{{\bar{N}}_{k}}$ by (25)–(30)13:**if** formation constraints enabled **then**14:   **Formation Optimization:** Perform gradient descent for all agents:$${\hat{p}}_{i}^{\left(k+1\right)}={\hat{p}}_{i}^{\left(k\right)}-\alpha \lambda_{2}\frac{\partial J_{f}}{\partial {\hat{p}}_{i}}{|}_{{\hat{p}}^{\left(k\right)}}$$15:   Repeat until convergence: ${∥{\hat{p}}^{\left(k+1\right)}-{\hat{p}}^{\left(k\right)}∥}_{F}<\epsilon_{\mathrm{tol}}$ or maximum iterations reached16:**end if**

**Remark** **2.** *The current formulation assumes a known, fixed formation geometry encoded in the distance matrix $D_{f}$ (defined in Section 3.3; ${\left[D_{f}\right]}_{ij}=d_{ij}^{f}$). For dynamic formation reconfiguration in real missions—e.g., when the swarm switches from one formation pattern to another—$D_{f}$ can be updated at each time step to reflect the new expected inter-agent distances; the algorithmic structure (GNA, listener localization, back calibration, formation optimization) remains applicable without modification. Thus, the method can adapt to time-varying formation geometry provided the updated $D_{f}$ is supplied (e.g., by a higher-level mission planner). Online estimation of time-varying formation geometry from measurements is beyond the scope of this work and is left for future study.*

## 4. Performance Analysis

This work seeks to analyze how formation constraints may improve localization accuracy in terms of information gain (relevant to backbone selection) and error propagation mitigation (relevant to listener localization and global optimization).

Formation constraints enhance localization by incorporating geometric prior information. The measurement objective function $J_{m}$ (defined in (7)) minimizes the squared errors between predicted and measured TOA and AOA values. The combined objective becomes

(37) $$J_{t}=J_{m}+\lambda J_{f},$$

where $J_{f}$ penalizes deviations from the expected formation geometry and λ>0 is the constraint weight.

### 4.1. Information Gain

The Fisher Information Matrix (FIM) quantifies the information that measurements provide about the unknown state θ (e.g., agent positions and orientations). The unconstrained FIM is

(38) $$F_{u}=E\left[\frac{\partial \log p(z|\theta )}{\partial \theta }\frac{\partial \log p(z|\theta )}{\partial \theta^{T}}\right],$$

where p(z|θ) is the measurement likelihood. With formation constraints, the effective FIM becomes

(39) $$F_{c}=F_{u}+\lambda F_{f},$$

where $F_{f}$ is the information contributed by the formation term $J_{f}$. The matrix $F_{f}$ is positive semi-definite, since it arises from the Hessian of a squared-distance penalty. Since $F_{f}$ is positive semi-definite, $\lambda F_{f}$ is also positive semi-definite for λ>0. Therefore, $F_{c}-F_{u}=\lambda F_{f}$ is positive semi-definite, which implies that $F_{c}$ provides no less information than $F_{u}$, thus increasing total information and lowering the Cramér-Rao Lower Bound (CRLB).

The improvement can be expressed via error covariance. The unconstrained covariance satisfies

(40) $$\Sigma_{u}\approx F_{u}^{-1},$$

while the constrained one satisfies

(41) $$\Sigma_{c}\approx {\left(F_{u}+\lambda F_{f}\right)}^{-1},$$

where the approximation holds when the FIM is well-conditioned. Because $F_{f}$ is positive semi-definite, $\mathrm{tr}\left(\Sigma_{c}\right)\leq \mathrm{tr}\left(\Sigma_{u}\right)$, thus the estimation error is reduced. The relative improvement depends on λ and the strength of the formation prior. A larger λ or a more informative formation structure yields a smaller $\mathrm{tr}(\Sigma_{c})$.

### 4.2. Error Propagation Mitigation

In cooperative localization, errors propagate along the dependency chain from backbone to listener agents. For a listener *k* with position ${\hat{p}}_{k}$ depending on backbone positions ${\left\{{\hat{p}}_{i}\right\}}_{i\in N_{b}}$,

(42) $$\Delta p_{k}\approx \sum_{i\in N_{b}}\frac{\partial p_{k}}{\partial p_{i}}\Delta p_{i}+n_{k},$$

where $\Delta p_{i}$ are backbone errors and $n_{k}$ is the error from listener measurements. Without constraints, the $\Delta p_{i}$ can be large and weakly correlated; thus, errors accumulate.

Formation constraints enforce geometric consistency. For agents with a formation relationship, $∥{\hat{p}}_{i}-{\hat{p}}_{j}∥\approx d_{ij}^{f}+\epsilon_{ij}$ with small $\epsilon_{ij}$. This ties the relative positions of neighboring agents to the prior $d_{ij}^{f}$, which bounds relative errors and reduces error accumulation along the chain. As a result, both backbone and listener estimates are pulled toward a geometrically consistent configuration.

**Remark** **3.** *Formation constraints act as a Tikhonov-type regularizer that guides the solution toward physically reasonable configurations. When λ is chosen appropriately, they may provide three benefits. (1) Ambiguity resolution: When the measurement model has multiple solutions (e.g., symmetric or degenerate geometries), the formation term prefers the one that matches the expected inter-agent distances, thus helping resolve ambiguities. (2) Outlier suppression: Large, isolated deviations from the formation are penalized by $J_{f}$, so estimates that would otherwise explain measurements through a few poor links are pulled back toward the prior geometry. (3) Noise robustness: Under strong measurement noise, $J_{m}$ alone can yield unstable or scattered estimates; the formation term stabilizes the objective and reduces the optimizer’s sensitivity to individual noisy measurements. These effects may moderately improve both accuracy and robustness.*

### 4.3. Complexity Analysis

In summary, the overall computational complexity of the proposed formation-constrained cooperative localization algorithm is given by $O\left(N^{2}N_{b}+N_{b}^{3}+N_{L}N_{b}+K_{\max}N^{2}\right)$, where *N* is the total number of UAV agents, $N_{b}$ is the number of backbone agents, $N_{L}=N-N_{b}$ denotes the number of listener agents, and $K_{\max}$ is the maximum number of iterations in the formation optimization stage.

Since *N* and $N_{b}$ are typically moderate in swarm deployment scenarios, with $N_{b}\ll N$ and $K_{\max}$ being a bounded constant, the lower-order term $O(N_{L}N_{b})$ from the WLS-based listener localization step is negligible in practice. The dominant computational costs, namely $O(N^{2}N_{b})$ for the formation-constrained GNA backbone selection, $O(N_{b}^{3})$ for backbone node localization, and $O(K_{\max}N^{2})$ for the global formation optimization refinement, remain computationally tractable for standard embedded or onboard UAV hardware. Thus, the proposed algorithm is feasible for real-time or near-real-time formation-constrained cooperative localization applications.

## 5. Numerical Experiments

This section presents numerical experiments to validate the effectiveness of the proposed formation-constrained cooperative localization method.

### 5.1. Experimental Setup

The simulation environment consists of a 10×10 m^2^ area with N=10 agents. TOA noise standard deviation: $\sigma_{\tau }=0.2/\left(3\times 10^{8}\right)$ s; AOA noise standard deviation: $\sigma_{\psi }=\pi /50$ rad. In the simulation of the localization algorithm, the experiments were performed across multiple formation types to evaluate the method’s adaptability. Each experiment consists of N=10 agents (We focus on small-scale UAV swarms in this work, but the proposed method can also be applied to large-scale UAV swarm localization by using the Distributed Geometry Merging approach from [27]) in total—$N_{b}=4$ backbone nodes and $N_{L}=6$ listener nodes—across 5000 Monte Carlo trials.

The formation geometries used in the localization simulation are defined as follows:

**Line + Wingman Formation:** This formation consists of N−1 agents arranged in a straight line with spacing *s*, and a single wingman agent positioned off the line:

$$\begin{array}{cc} p_{i}^{f} & ={\left[\left(i-1\right)\cdot s,0\right]}^{T},\;i=1,\ldots ,N-1,\\ p_{N}^{f} & ={\left[3s,1.5\right]}^{T}, \end{array}$$

where s=3.0 m. This formation is commonly used in convoy protection and patrol missions, where the main line provides forward coverage while the wingman offers lateral surveillance and early threat detection.

**Grid Formation:** This formation consists of agents arranged in a rectangular grid with uniform spacing *s* in both *x* and *y*. The grid has $C=\lceil \sqrt{N}\rceil$ columns and R=⌈N/C⌉ rows, with nodes indexed in row-major order:

$$p_{i}^{f}={\left[\left(c-1\right)\cdot s,\;\left(r-1\right)\cdot s\right]}^{T},$$

where r=⌊(i−1)/C⌋+1 is the row index, c=mod(i−1,C)+1 is the column index, and s=2.0 m. The grid formation provides regular spatial distribution suitable for area coverage, distributed sensing, and coordinated inspection tasks where uniform node spacing is desired.

**Diamond Formation:** This formation consists of agents on the four edges of a diamond. The four corner nodes and six edge nodes are:

$$\begin{array}{cc} p_{1}^{f} & ={\left[0,R_{o}\right]}^{T},\;p_{2}^{f}={\left[R_{o},0\right]}^{T};\\ p_{3}^{f} & ={\left[0,-R_{o}\right]}^{T},\;p_{4}^{f}={\left[-R_{o},0\right]}^{T};\\ p_{5}^{f} & ={\left[R_{o}/3,2R_{o}/3\right]}^{T},\;p_{6}^{f}={\left[2R_{o}/3,-R_{o}/3\right]}^{T};\\ p_{7}^{f} & ={\left[R_{o}/3,-2R_{o}/3\right]}^{T},\;p_{8}^{f}={\left[-R_{o}/3,-2R_{o}/3\right]}^{T};\\ p_{9}^{f} & ={\left[-2R_{o}/3,R_{o}/3\right]}^{T},\;p_{10}^{f}={\left[-R_{o}/3,2R_{o}/3\right]}^{T}. \end{array}$$

where $R_{o}=3.0$ m. The corners (1–4) are top, right, bottom, and left; nodes 5–10 lie on the edges. The diamond formation provides 360-degree coverage suitable for defense and escort missions.

**Circular Formation:** This formation consists of agents uniformly distributed on a circle:

$$\varphi_{i}=\frac{2\pi (i-1)}{N},\;p_{i}^{f}=R\cdot {\left[\cos\left(\varphi_{i}\right),\sin\left(\varphi_{i}\right)\right]}^{T},\;i=1,\ldots ,N,$$

where $\varphi_{i}$ is the angular position of agent *i* on the circle (distinct from the state $\theta_{i}$ in Section 2.2) and R=3.0 m is the circle radius. The circular formation provides symmetric geometric distribution and is typically employed in perimeter surveillance, area monitoring, and coordinated search operations where equal coverage in all directions is required.

The following metrics are used to evaluate localization performance.

**Mean/Median RMSE (over trials):**

$$\begin{array}{cc} {\mathrm{RMSE}}_{\mathrm{mean}} & =\frac{1}{N_{\mathrm{trials}}}\sum_{t=1}^{N_{\mathrm{trials}}}\sqrt{\frac{1}{N}\sum_{i=1}^{N}{∥{\hat{p}}_{i}^{\left(t\right)}-p_{i}^{\mathrm{true}}∥}^{2}},\\ {\mathrm{RMSE}}_{\mathrm{median}} & =\mathrm{median}\left({\left\{\sqrt{\frac{1}{N}\sum_{i=1}^{N}{∥{\hat{p}}_{i}^{\left(t\right)}-p_{i}^{\mathrm{true}}∥}^{2}}\right\}}_{t=1}^{N_{\mathrm{trials}}}\right). \end{array}$$

The median RMSE provides a robust metric that is less sensitive to outliers compared to the mean RMSE.

### 5.2. Performance of Formation-Constrained GNA

**Figure 2:**
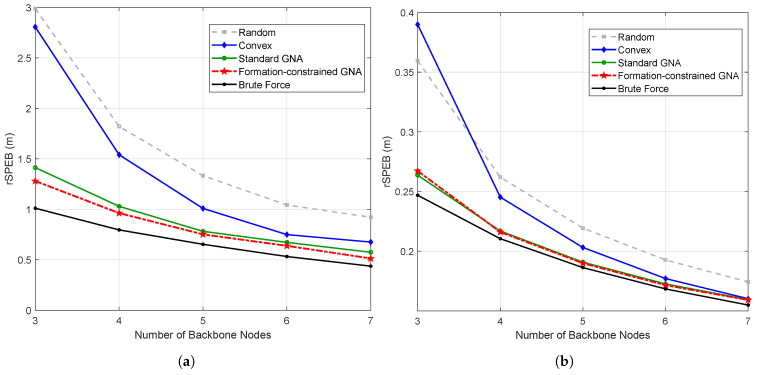

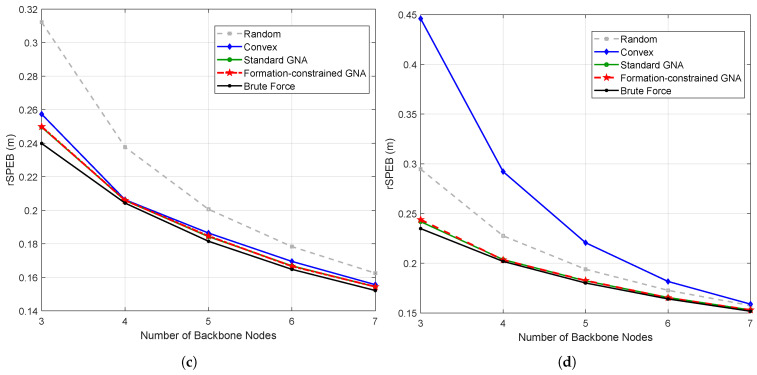
Root SPEB comparison of different backbone node selection strategies in different formations: (**a**) line + wingman formation; (**b**) grid formation; (**c**) diamond formation; (**d**) circular formation.

The performance of the proposed formation-constrained backbone selection strategy is compared with several benchmark methods, as illustrated in Figure 2. The random strategy (marked as squares) selects backbone agents uniformly at random from all agents. The convex strategy (marked as diamonds) selects backbone agents on the convex hull of the agent positions. The standard GNA strategy (marked as circles) applies the reference method [27] without using formation information. The formation-constrained GNA strategy (marked as pentagrams) applies Algorithm 1. Finally, the brute force solution (marked as stars) is obtained by exhaustively traversing all candidate backbone sets and choosing the one that minimizes the theoretical rSPEB (root squared position error bound) [31].

As shown in Figure 2, for highly regular formations such as circular, grid, and diamond, the standard GNA strategy already yields backbone sets that are very close to the brute force solution. As a result, the proposed formation-constrained GNA provides only marginal improvement in the theoretical rSPEB metric in these symmetric cases. This behavior is consistent with the intuition that, when the node geometry is well-conditioned and almost symmetric, there is limited room for further improvement in terms of Fisher information. In contrast, for irregular and asymmetric “line + wingman” formations, the standard GNA may select suboptimal backbones, while the proposed formation-constrained GNA can better exploit the known formation pattern and achieve a more significant reduction in rSPEB.

### 5.3. Performance of Formation-Constrained Cooperative Localization

**Figure 3:**
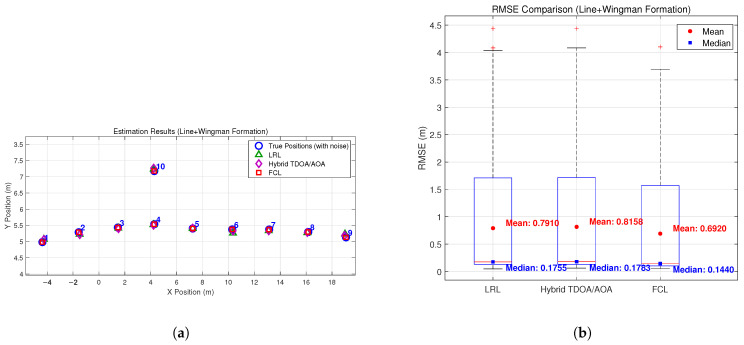
Estimation results: (**a**) estimation results in the Line + Wingman formation; (**b**) RMS errors of the Line + Wingman formation.

**Figure 4:**
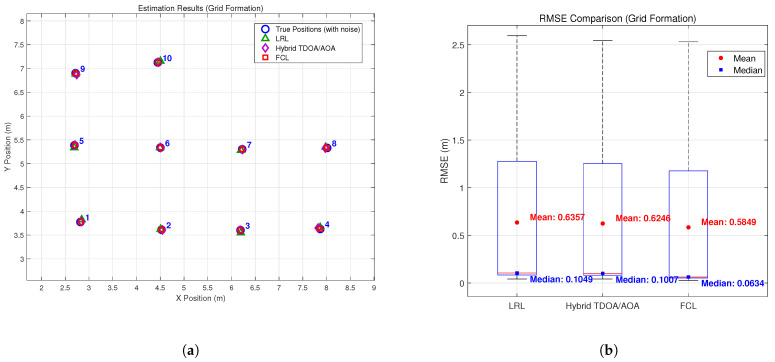
Estimation results: (**a**) estimation results in the Grid formation; (**b**) RMS errors of the Grid formation.

**Figure 5:**
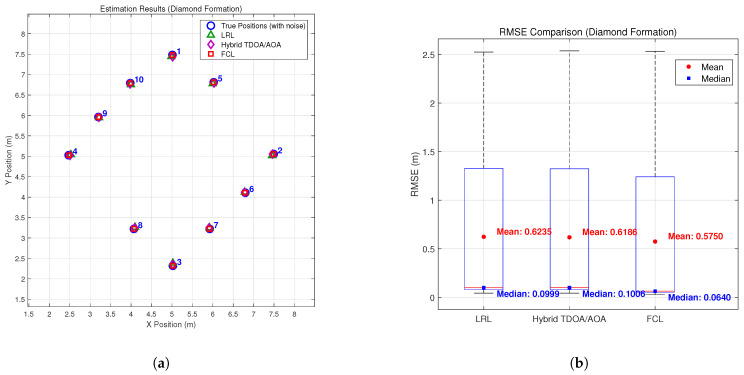
Estimation results: (**a**) estimation results in the diamond formation; (**b**) RMS errors of the diamond formation.

**Figure 6:**
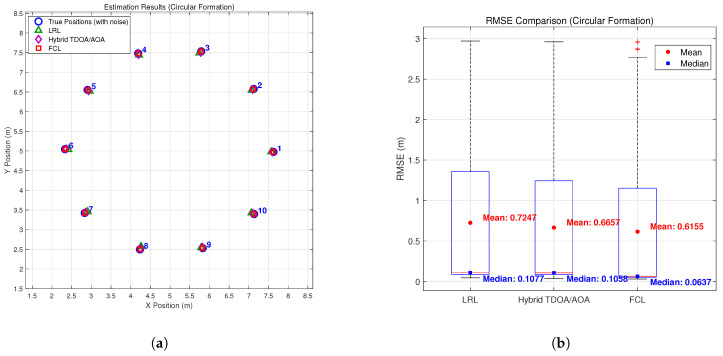
Estimation results: (**a**) estimation results in the circular formation; (**b**) RMS errors of the circular formation.

To validate the effectiveness of the proposed method, we conducted simulations under multiple typical formation configurations. Figure 3, Figure 4, Figure 5 and Figure 6 compare the estimation performance and RMSE of the proposed formation-constrained localization (FCL) method with those of two benchmark methods: (1) the listener relative localization (LRL) method reported in [27], and (2) the hybrid TDOA/AOA localization method [17], which adopts the same GNA-based backbone node selection strategy as the scheme proposed in [27]. Neither benchmark exploits formation geometry. For the box plots, the median is marked by the red line, the interquartile range by the blue box, the whiskers by black dashed lines, and outliers by red crosses. Additional statistical markers, including the mean (red circles) and median (blue squares) of the full dataset, are provided with corresponding numerical annotations.

The simulation results demonstrate that the proposed FCL method consistently outperforms both the LRL and the hybrid TDOA/AOA benchmarks across all tested formation types. The hybrid TDOA/AOA method, although it fuses TOA and AOA measurements, does not use formation constraints; its RMSE is comparable to or slightly higher than LRL in most formations, and both are clearly outperformed by FCL. This indicates that integrating formation geometry into the localization process yields measurable gains over methods that use the same backbone selection but lack formation constraints. While the theoretical rSPEB analysis shows only modest gains for symmetric formations, the full localization experiments show that the proposed formation-constrained method consistently reduces the RMSE compared with both benchmark methods. Beyond the information-theoretic lower bound, formation constraints help stabilize the non-linear estimation process and mitigate error propagation in practice.

Improvements are relatively significant in the line + wingman formation, where both LRL and the hybrid TDOA/AOA method exhibit clear deviations for wingman nodes, while the proposed FCL method locates both the main line and wingman nodes more closely. The proposed method also features a narrower, lower-shifted RMSE and fewer large errors than both benchmarks. For the diamond, grid, and circular formations, the proposed method consistently outperforms LRL and the hybrid TDOA/AOA method: it preserves the original formation structure, its estimates gather closely around the true positions, and it achieves a tighter, lower RMSE distribution for enhanced consistency and stability.

Table 2 presents the single-trial runtime of all compared methods under different formations, averaged over 5000 Monte Carlo runs. All timings were obtained using MATLAB R2022b on a PC equipped with a 13th Gen Intel Core i9-13900HX processor (2.20 GHz) and 64 GB RAM. The results show that the proposed FCL method maintains millisecond-level runtime per trial across all tested formation configurations, with only a marginal increase compared with the benchmark LRL and Hybrid TDOA/AOA methods, and fully meets the real-time requirements of cooperative formation localization systems.

## 6. Conclusions

This work seeks to establish a formation-constrained cooperative localization method for UAV swarms in GNSS-denied environments. It aims to systematically integrate known formation geometry into localization processes. First, the formation-constrained GNA strategy helps improve measurement geometry quality by integrating formation geometry into backbone selection. Second, the formation-constrained listener localization reduces ambiguity and enhances accuracy through explicit constraint integration during position estimation. Third, the global optimization stage helps maintain geometric consistency across all agents, mitigating error propagation. Simulation results of the localization process indicate the method achieves good performance in success rate, stability, and accuracy and performs stably across different formation types. The experiments across line + wingman, grid, diamond, and circular formations illustrate the method’s performance under different single-task formation patterns. In addition, its ability to adapt to asymmetric geometries (line + wingman) highlights its potential practical value for applications such as tunnel and corridor operations. Potential extensions include applying the proposed method to three-dimensional (3D) UAV formations and to online estimation of time-varying formation geometry; the integration of multi-task collaboration with formation-constrained localization also merits further investigation. Generalizing the approach to 3D would require elevation-capable AOA measurements, at least four non-coplanar anchors for backbone localization, and careful treatment of vertical observability when formations are coplanar or near-planar; addressing these aspects would strengthen the applicability of formation-constrained localization to full 3D swarm operations and is left for future work.

## Figures and Tables

**Table 1:** Mathematical  notations.

Notation	Definition
$X^{T},X^{-1}$	Transpose and inverse of X
${\left[x\right]}_{k:j}$	A sub-vector consisting of the *k*th to *j*th elements of x
${\left[X\right]}_{i:j,m:n}$	A sub-matrix of X spanning rows *i* to *j* and columns *m* to *n*
$I_{n},1_{n},0_{n}$	*n*-dimensional identity matrix, all-ones vector, and all-zeros vector
$E_{x}\left\{\cdot \right\}$	Expectation operator over the random variable x
${∥x∥}_{F}$	Frobenius norm of x (Euclidean norm when x is a vector)
⊙,⊗	Hadamard product and Kronecker product operator
tr{·}	Trace operator
diag{x}	Diagonal matrix operator using elements in x
|N|	Cardinality of the set N

**Table 2:** Average Runtime per Trial (s) Under Different Formations.

Method	Line + Wingman	Diamond	Grid	Circular
LRL	0.0081	0.0108	0.0221	0.0122
Hybrid TDOA/AOA	0.0080	0.0108	0.0217	0.0122
FCL	0.0087	0.0119	0.0237	0.0134

## Data Availability

The original contributions presented in this study are included in the article. Further inquiries can be directed to the corresponding author.

## Abbreviations

The following abbreviations are used in this manuscript:

GNSS	Global Navigation Satellite System
UAV	Unmanned Aerial Vehicle
UWB	Ultra-Wideband
TOA	Time of Arrival
TDOA	Time Difference of Arrival
AOA	Angle of Arrival
MDS	Multidimensional Scaling
GNA	Greedy Node Activation
NLOS	Non-Line-of-Sight
FIM	Fisher Information Matrix
CRLB	Cramér-Rao Lower Bound
MSE	Mean Squared Error
RMSE	Root Mean Square Error
rSPEB	(root Squared Position Error Bound)
